# Supplementary material for: Light-switchable transcription factors obtained by direct screening in mammalian cells
Source: Nat Commun. 2023 Jun 2;14:3185. doi: 10.1038/s41467-023-38993-6 (PMC10238501; doi:10.1038/s41467-023-38993-6)
Supplement: Supplementary file 1 — Supplementary Information [file 41467_2023_38993_MOESM1_ESM.docx]

Supplementary Materials for

Light-switchable transcription factors obtained by direct screening in mammalian cells

Liyuan Zhu, Harold M. McNamara, Jared E. Toettcher

Correspondence to: [toettcher@princeton.edu](mailto:xxxxx@xxxx.xxx)

**This PDF file includes:**

Supplementary Tables 1-5

Supplementary Figures 1-9

Supplementary Notes 1-4

**Supplementary Tables**

**Supplementary Table 1: Coverage fold quantification of transposition**

| Colony forming unit | Plasmid length (bp) | Coverage fold calculation |
| --- | --- | --- |
| 1.14x10^6^ | 2951 | $\frac{1.14\times{10}^{6}}{2\times2951}=193$ |

**Supplementary Table 2: Plasmids constructed for this study**

| **Plasmids** | **Insert1** | **Insert2** | **Vector** | **Source** |
| --- | --- | --- | --- | --- |
| pATT-Dest | Empty |  | pUC19 | Addgene #79770 |
| pUCKanR-Mu-BsaI | Mu-BsaI |  | pUC19 | Addgene #79769 |
| pLZA066 | Gal4-VP64 |  | pUC19 | This study |
| pLZA042 | 5×UAS_dGFP | PGK_iRFP | pHR | Pavithran et al. |
| pJG75 | chicken beta-actin promoter_Bxb1 Integrase |  |  | Matreyek et al. |
| pJG82 | mCherry |  | AttB recombination | Matreyek et al. |
| pJG83 | EGFP |  | AttB recombination | Matreyek et al. |
| pIRES2-mCherry-p53 deltaN | p53 deltaN |  | pIRES2-EGFP | Addgene #49243 |
| pLZA063 | Gal4-VP64-IRES-mCherry |  | AttB recombination | This study |
| pLZA107 | Gal4LOV^SK22^-VP64-IRES-mCherry |  | AttB recombination | This study |
| pLZA139 | SFFV_Gal4-VP64-IRES-mCherry |  | pHR | This study |
| pLZA140 | SFFV_Gal4LOV^SK22^-VP64-IRES-mCherry |  | pHR | This study |
| pLZA141 | SFFV_Gal4LOV^KN23^-VP64-IRES-mCherry |  | pHR | This study |
| pLZA142 | SFFV_Gal4LOV^AK29^-VP64-IRES-mCherry |  | pHR | This study |
| pLZA120 | T7_Gal4_Histag |  | PET | This study |
| pLZA121 | T7_Gal4LOV^SK22^_Histag |  | PET | This study |
| pVITRO1-SS-113 | EF-1α_ZF9-VP64 | CAG_mCherry | pVITRO1-NEO-MCS | Addgene #68737 |
| pLZA125 | EF-1α_ZF9LOV^C1^-VP64 | CAG_mCherry | pVITRO1-NEO-MCS | This study |
| pLZA126 | EF-1α_ZF9LOV^C2^-VP64 | CAG_mCherry | pVITRO1-NEO-MCS | This study |
| pLZA127 | EF-1α_ZF9LOV^C3^-VP64 | CAG_mCherry | pVITRO1-NEO-MCS | This study |
| pLZA128 | EF-1α_ZF9LOV^C4^-VP64 | CAG_mCherry | pVITRO1-NEO-MCS | This study |
| pGL4.26-SS-192 | ZF9operon×6_GFP |  | pGL4.26 luc2/minp/hygro | Addgene #68759 |
| pCMV-QF | CMV_QF |  | pcDNA3.1 | Addgene #24339 |
| pLZA133 | CMV_QFLOV^DG87^ |  | pcDNA3.1 | This study |
| pLZA134 | CMV_QFLOV^GI88^ |  | pcDNA3.1 | This study |
| pLZA137 | CMV_QFLOV^VS97^ |  | pcDNA3.1 | This study |
| pQUAS-luc2 | QUAS_luc2 |  | pGL4.23 | Addgene 24337 |
| pLZA138 | QUAS_dGFP |  | pGL4.23 | This study |
| pLZA144 | SFFV_Gal4LOV^SK22^-VP64-mCherry |  | pHR | This study |

**Supplementary Table 3: Plasmid random insertion library constructed for this study**

| **Name** | **Original plasmid** | **Random insertion elements** | **Insertion region** |
| --- | --- | --- | --- |
| pLZA066_CmR01 | pLZA066 | Chloramphenicol-resistant gene | Whole plasmid |
| pLZA066_LOV01 | pLZA066 | AsLOV2(408-543) (0 reading frame) | Whole plasmid |
| pLZA066_LOV02 | pLZA066 | AsLOV2(408-543) (+1 reading frame) | Whole plasmid |
| pLZA066_LOV03 | pLZA066 | AsLOV2(408-543) (+2 reading frame) | Whole plasmid |
| pLZA063_LOV01 | pLZA063 | AsLOV2(408-543) (0 reading frame) | Gal4-VP64 coding region |
| pLZA063_LOV02 | pLZA063 | AsLOV2(408-543) (+1 reading frame) | Gal4-VP64 coding region |
| pLZA063_LOV03 | pLZA063 | AsLOV2(408-543) (+2 reading frame) | Gal4-VP64 coding region |

**Supplementary Table 4: Stable cell line used for this study**

| **Name** | **Description** |
| --- | --- |
| 293T LP | 293T cells with a single landing pad sequence incorporated |
| 293T LPR | 293T LP cells with UAS-dGFP sequence incorporated with lentivirus |
| 293T LPR + pLZA063 | 293T LP UAS-dGFP cells with Gal4-VP64-IRES-mCherry integrated into landing pad locus |
| 293T LPR + pLZA107 | 293T LP UAS-dGFP cells with Gal4LOV^SK22^-VP64-IRES-mCherry integrated into landing pad locus |

**Supplementary Table 5: Cell libraries used for selections**

| **Name** | **Description** |
| --- | --- |
| 293-LPR + LZA063_LOV01 | 293T LP UAS-dGFP with Gal4-VP64-IRES-mCherry integrated into landing pad locus where Gal4-VP64 is randomly inserted with AsLOV2(408-543) (0 reading frame) |
| 293T LPR + LZA063_LOV02 | 293T LP UAS-dGFP with Gal4-VP64-IRES-mCherry integrated into landing pad locus where Gal4-VP64 is randomly inserted with AsLOV2(408-543) (+1 reading frame) |
| 293T LPR + LZA063_LOV03 | 293T LP UAS-dGFP with Gal4-VP64-IRES-mCherry integrated into landing pad locus where Gal4-VP64 is randomly inserted with AsLOV2(408-543) (+2 reading frame) |
| 293T LPR + LZA063_LOV01_S1 | Sorted mCherry+ BFP- population of 293T LP UAS-dGFP + LZA063_LOV01 |
| 293T LPR + LZA063_LOV02_S1 | Sorted mCherry+ BFP- population of 293T LP UAS-dGFP + LZA063_LOV02 |
| 293T LPR + LZA063_LOV03_S1 | Sorted mCherry+ BFP- population of 293T LP UAS-dGFP + LZA063_LOV03 |
| 293T LPR + LZA063_LOV02_S2 | Sorted high GFP population in dark of 293T LP UAS-dGFP + LZA063_LOV02_S1 |
| 293T LPR + LZA063_LOV02_S3 | Sorted low GFP population in light of 293T LP UAS-dGFP + LZA063_LOV02_S2 |
| 293T LPR + LZA063_LOV02_S6 | Cells recovered after three times dark and twice light GFP sorting of 293T LP UAS-dGFP + LZA063_LOV02_S1 |
| 293T LPR + LZA063_LOV02_S7 | Cells recovered after sorting the small amount of high GFP population only present in dark of 293T LP UAS-dGFP + LZA063_LOV02_S6 |

**Supplementary Figures**


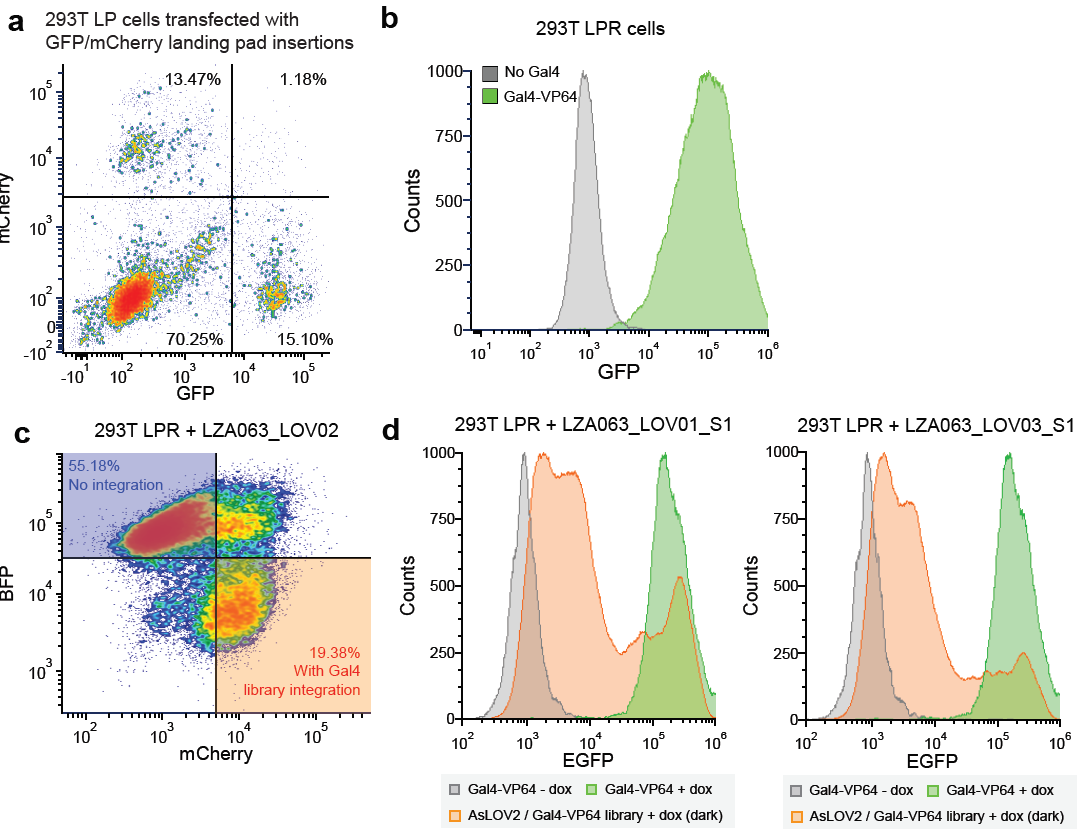


**Supplementary Figure 1. Verification of reporter cell line and integration of library into reporter cell line** (**a**) Transfection with a combination of GFP and mCherry integration cassettes to landing pad cell line resulted in either GFP+ or mCherry+ cells, indicative of a single integration per cell. (**b**) Wild type Gal4 could induce GFP expression in the 293T LP UAS-dGFP reporter cell line. (**c**) mCherry and BFP distribution of reporter cells directly after library integration, and the mCherry+/BFP- quadrant corresponds to cells with integration which were sorted for following selections. d. single cell GFP distribution of cells expressing LZA063_LOV01 and LZA063_LOV03. Source data for **a**~**d** are provided as a Source Data file


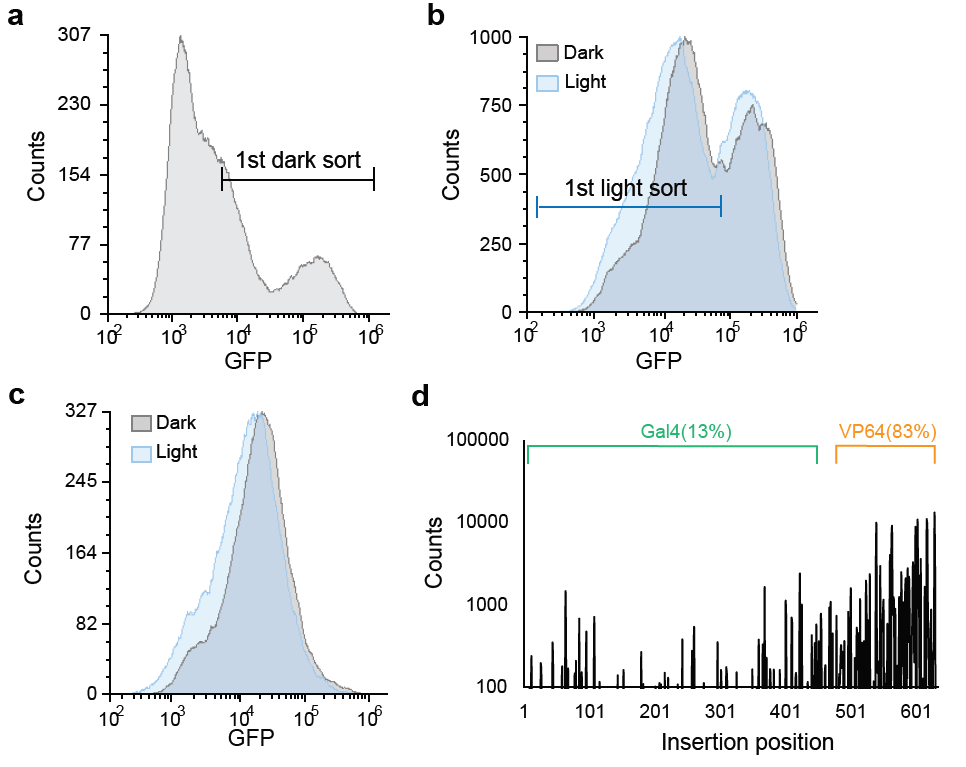


**Supplementary Figure 2. Details of selections for photoswitchable Gal4-VP64 variants.** (**a**) Cell populations with high GFP in dark in the initial library was first sorted. (**b**) After the 1st dark sort, cells exhibiting low GFP under light was then sorted. (**c**) single-cell GFP distribution in dark or light condition after one round of dark and light sort. (**d**) AsLOV2 insertion distribution of library after the first round of dark sort, with most of the insertions enriched in VP64, either in-frame or out-of-frame. When making the plot, 1 is added to the count number in all positions deliberately to accommodate the positions with 0 count in logarithm-scale plot. Raw sequencing data was available in SRA with the accession code SAMN35215820 [https://www.ncbi.nlm.nih.gov/biosample/35215820]. Source data for **a**~**d** are provided as a Source Data file.


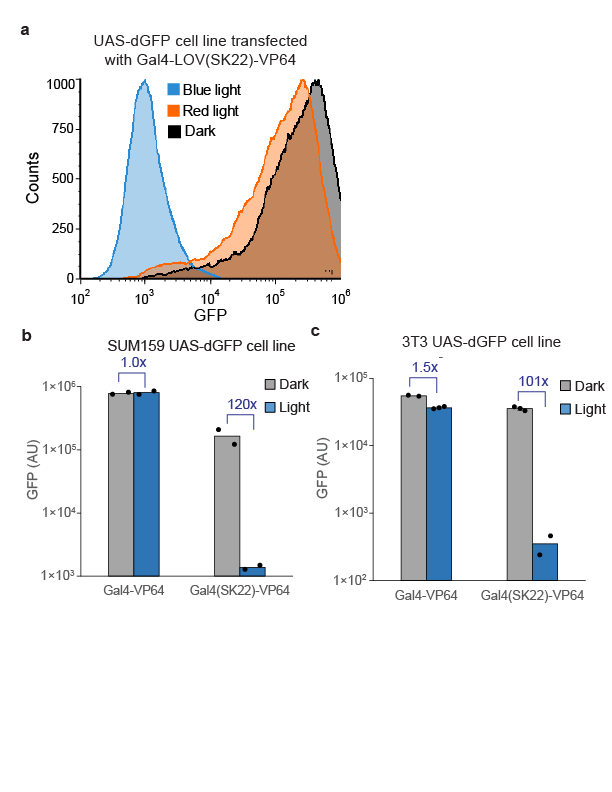


**Supplementary Figure 3. Additional characterization of LightsOut system and its variants.** (**a**). Cells expressing LightsOut Gal4-VP64 transcription factor were illuminated with an equivalent red-light source for 24 h and compared to dark or blue light illumination to test whether the light-switchable response results from general phototoxicity or heat produced from the light source. (**b**). Light-dependent GFP expression of SUM159 UAS-dGFP cell line transfected with unmodified Gal4-VP64 or LightsOut tool. Cells were incubated in dark or light for 22 h before GFP measurement by flow cytometry. Two biological replicates were measured for each condition, and the error bars indicate mean ± SEM (**c**). Light-dependent GFP expression of 3T3 UAS-dGFP cell line transfected with unmodified Gal4-VP64 or LightsOut tool. Cells were incubated in dark or light for 22 h before GFP measurement by flow cytometry. Three biological replicates for Gal4-VP64 in light and Gal4(SK22)-VP64 in dark conditions, two biological replicates for other conditions were measured. Error bars indicated (where n>2) mean ± SEM. Source data for **a**~**d** are provided as a Source Data file


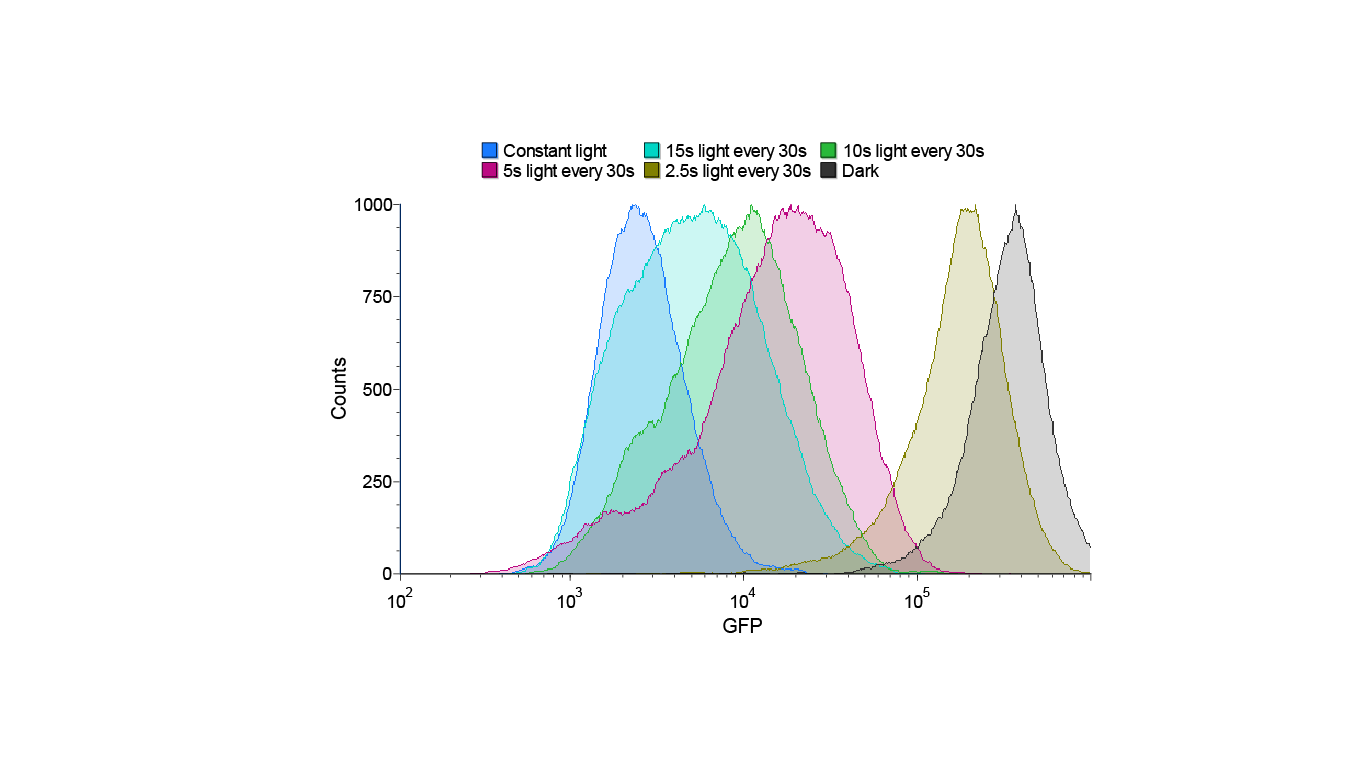


**Supplementary Figure 4. Raw dose response profiles for the LightsOut system.** Flow cytometry histograms collected for 293-LPR cells harboring Gal4LOV^SK22^-VP64 and treated with different pulse sequences of light. Data is from one biological replicate of the results shown in **Figure 3c**. Source data are provided as a Source Data file


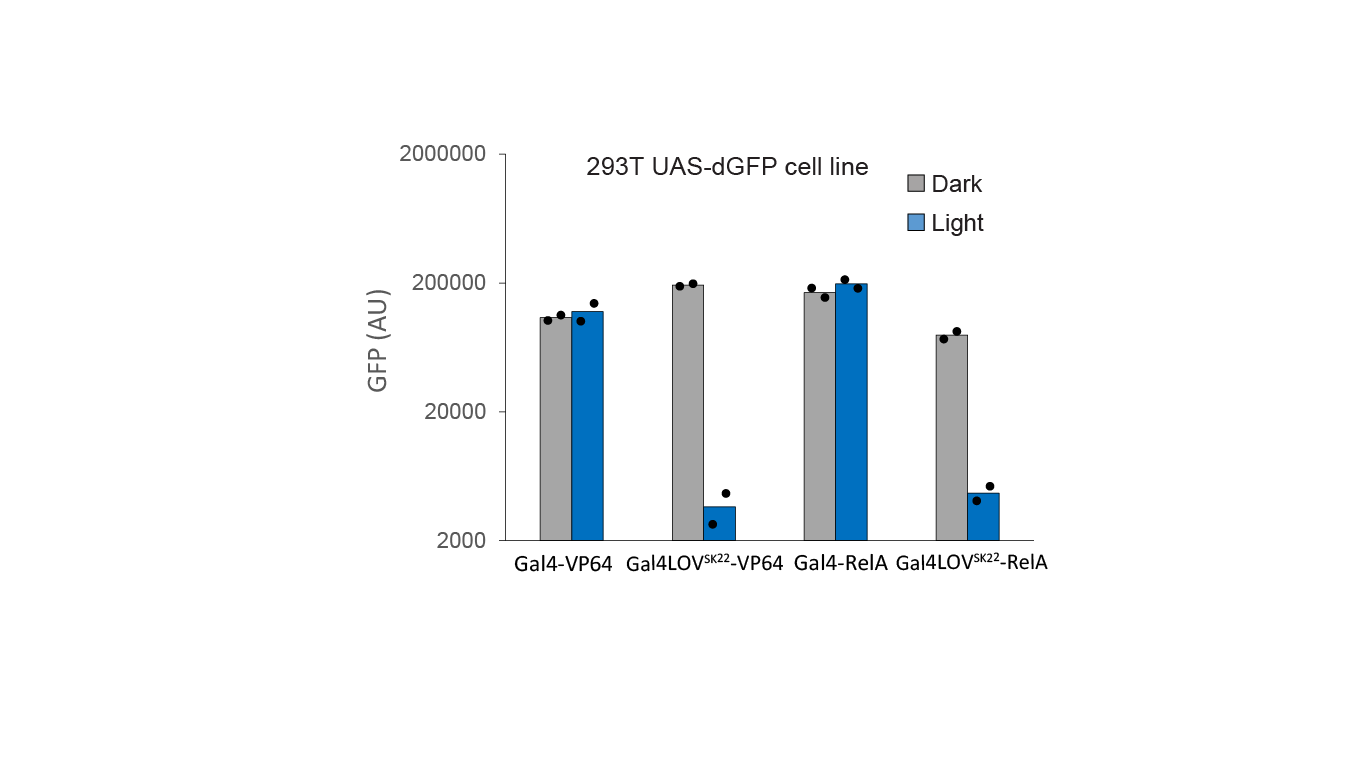


**Supplementary Figure 5. Test LightsOut with different transactivation domain.** Light-dependent GFP expression of 293T UAS-dGFP cell line transfected with Gal4-RelA or Gal4LOV^SK22^-RelA, as well as unmodified Gal4-VP64 or LightsOut tool (Gal4LOV^SK22^-VP64) for comparison. Cells were incubated in dark or light for 22 h before GFP measurement by flow cytometry. Two replicates were measured for each condition. Source data are provided as a Source Data file.


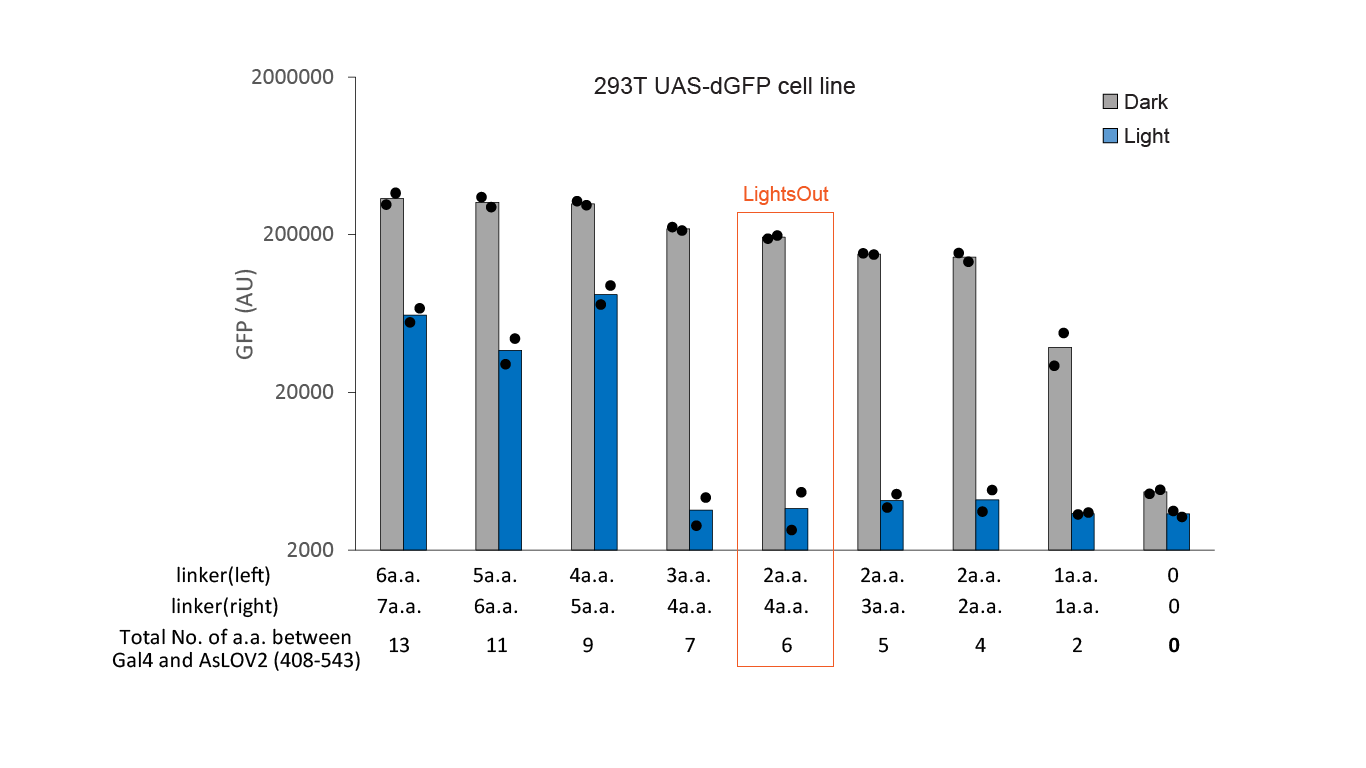


**Supplementary Figure 6. Test LightsOut with different linker lengths.** nine different Gal4LOV^SK22^ variants with a variety of linker lengths between AsLOV2(408-543) and Gal4 ranging from 0 amino acid (no linker) to 13 amino acids were tested, within which our engineered LightsOut corresponds to the variant whose linker length is 6 amino acids. After transfections, cells were incubated in dark or light for 22 h before GFP measurement by flow cytometry. Two replicates were measured for each condition. Source data are provided as a Source Data file.


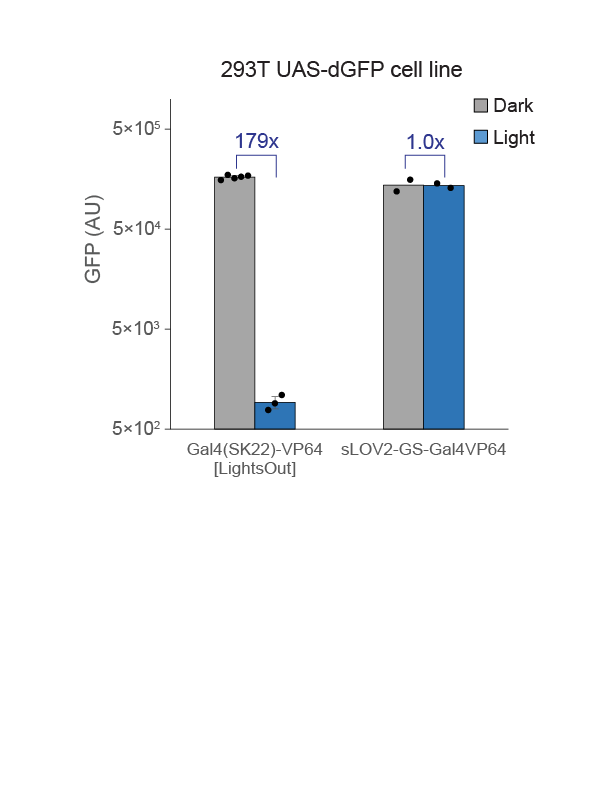


**Supplementary Figure 7. Testing for photoswitchable GFP in an N-terminal AsLOV2-Gal4 fusion.** 293 UAS-dGFP cells were transfected with AsLOV2(408-543)-GS-Gal4-VP64 (short LOV2 directly fused to Gal4-VP64 with two amino acids linker) or the LightsOut system as a control. Cells were incubated in dark or light for 22 h before GFP measurement by flow cytometry. Five biological replicates for Gal4(SK22)-VP64 in dark, three biological replicates for Gal4(SK22)-VP64 in light, two biological replicates for the rest conditions were measured. Error bars (where n>2) indicate mean ± SEM. Source data are provided as a Source Data file.


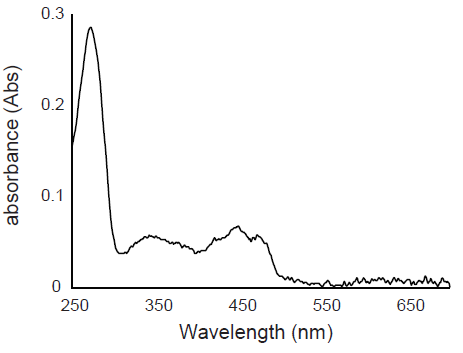


**Supplementary Figure 8. UV-Vis absorbance spectrum of purified Gal4LOV^SK22^ ranging from 250nm to 700nm.** Characteristic absorbance peak at 450 nm indicates incorporation of functional AsLOV2. Source data are provided as a Source Data file.


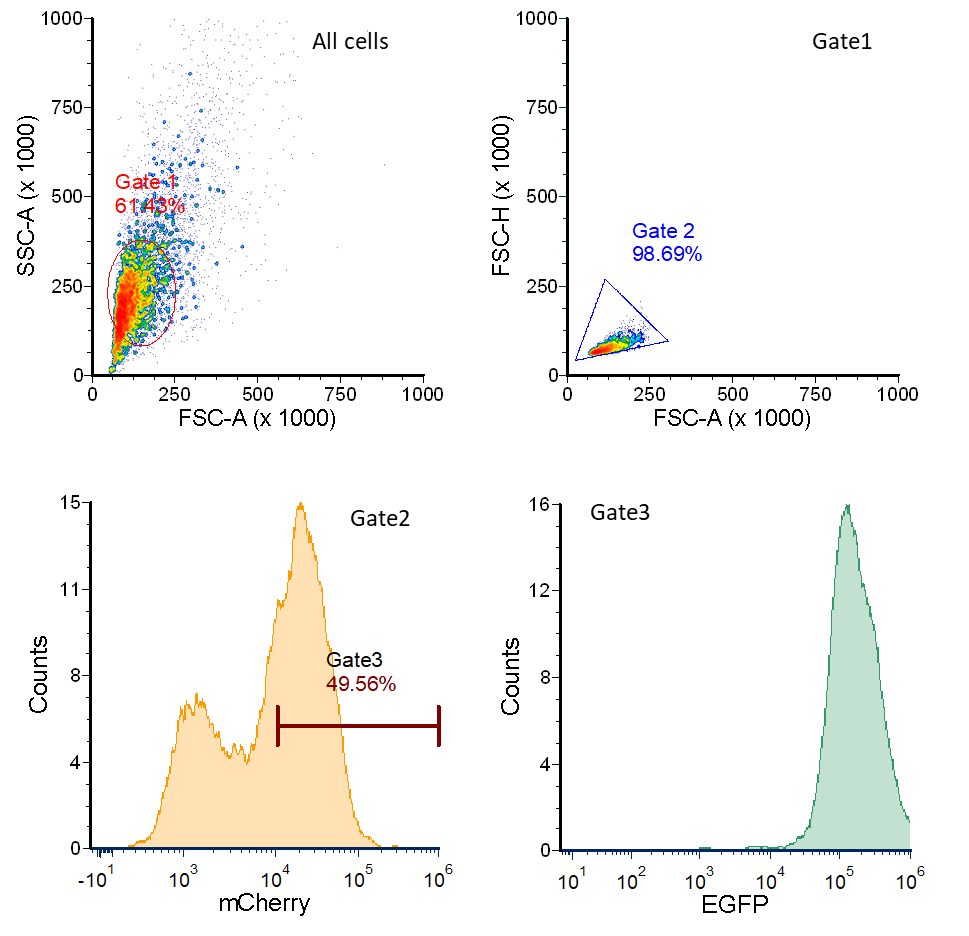


**Supplementary Figure 9. Gating strategy for flow cytometry.** FSC vs SSC plot was gated first for the exclusion of debris and dead cells and singlets were then identified using FSC-A versus FSC-H plot. If there is mCherry marker indicating expression of protein of interest then mCherry positive population was further gated in mCherry histogram plot.

**Supplementary Notes**

**Supplementary note 1: Gene sequences of *Gal4LOV^SK22^-VP64***

In black: Gal4(1-147)

In red: VP64

In green: Linker

In blue: AsLOV2 (408-543)

ATGAAGCTGCTGAGCAGCATCGAGCAGGCCTGTGACATCTGCCGGCTGAAGAAACTGAAGTGCAGTGCATCTTTGGAACGTATCGAAAAGAATTTTGTCATCACGGATCCGCGTCTTCCCGACAATCCGATTATCTTCGCGTCAGACTCTTTCTTACAACTGACTGAGTATAGTAGAGAGGAGATATTGGGGCGTAACTGTAGATTTCTTCAGGGGCCAGAAACTGATCGGGCTACCGTTCGCAAGATACGTGACGCAATAGACAACCAGACCGAGGTGACGGTGCAGCTGATTAACTACACAAAGTCTGGGAAGAAGTTCTGGAACCTGTTTCATTTACAACCTATGAGAGATCAAAAAGGTGACGTTCAATATTTCATCGGGGTTCAGTTAGATGGGACTGAGCACGTGAGAGATGCAGCAGAAAGAGAGGGTGTAATGCTTATTAAAAAAACAGCCGAGAATATCGACGAAGCCGCTGCGTCATGCAGCAAAGAAAAGCCCAAGTGCGCCAAGTGCCTGAAGAACAACTGGGAGTGCCGGTACAGCCCCAAGACCAAGAGAAGCCCCCTGACCAGAGCCCACCTGACCGAGGTGGAAAGCCGGCTGGAAAGACTGGAACAGCTGTTTCTGCTGATCTTCCCACGCGAGGACCTGGACATGATCCTGAAGATGGACAGCCTGCAGGACATCAAGGCCCTGCTGACCGGCCTGTTCGTGCAGGACAACGTGAACAAGGACGCCGTGACCGACAGACTGGCCAGCGTGGAAACCGACATGCCCCTGACCCTGCGGCAGCACAGAATCAGCGCCACCAGCAGCAGCGAGGAAAGCAGCAACAAGGGCCAGCGGCAGCTGACAGTGTCTGCTGCTGCAGGCGGAAGCGGAGGCTCTGGCGGATCTGATGCCCTGGACGACTTCGACCTGGATATGCTGGGCAGCGACGCCCTGGATGATTTTGATCTGGACATGCTGGGATCTGACGCTCTGGACGATTTCGATCTCGACATGTTGGGATCAGATGCACTGGATGACTTTGACCTGGACATGCTCGGATCA

**Supplementary note 2: Gene sequences of *QFLOV^DG87^***

In black: QF (1-816)

In green: Linker

In blue: AsLOV2 (408-543)

ATGCCGCCTAAACGCAAGACACTCAATGCCGCTGCCGAAGCCAATGCCCACGCTGATGGCCATGCTGATGGCAATGCTGATGGTCATGTCGCTAACACTGCAGCAAGCAGCAACAACGCCCGTTTTGCGGACTTGACCAACATTGACACACCCGGCCTCGGCCCTACCACGACGACGTTACTTGTCGAGCCCGCTCGTTCGAAACGCCAGAGAGTCTCGAGGGCCTGTGATCAGTGTCGAGCTGCACGTGAAAAGTGTGATGCATCTTTGGAACGTATCGAAAAGAATTTTGTCATCACGGATCCGCGTCTTCCCGACAATCCGATTATCTTCGCGTCAGACTCTTTCTTACAACTGACTGAGTATAGTAGAGAGGAGATATTGGGGCGTAACTGTAGATTTCTTCAGGGGCCAGAAACTGATCGGGCTACCGTTCGCAAGATACGTGACGCAATAGACAACCAGACCGAGGTGACGGTGCAGCTGATTAACTACACAAAGTCTGGGAAGAAGTTCTGGAACCTGTTTCATTTACAACCTATGAGAGATCAAAAAGGTGACGTTCAATATTTCATCGGGGTTCAGTTAGATGGGACTGAGCACGTGAGAGATGCAGCAGAAAGAGAGGGTGTAATGCTTATTAAAAAAACAGCCGAGAATATCGACGAAGCCGCTGCGTCAGGAATCCAGCCGGCTTGCTTCCCCTGTGTGTCGCAGGGCCGGTCGTGTACCTACCAGGCCAGTCCCAAGAAGCGAGGAGTCCAGACGGGCTACATCCGCACTCTCGAACTGGCTCTGGCTTGGATGTTCGAGAACGTTGCCCGCAGCGAGGACGCCCTCCACAATCTTTTGGTCCGTGATGCTGGCCAGGGCAGCGCTCTCCTGGTCGGCAAAGACTCGCCTGCTGCAGAACGCCTGCATGCAAGATGGGCGACGAGTCGAGTCAACAAAAGCATCACCCGTCTTCTCTCAGGTCAGGCCGCACAAGATCCATCTGAAGACGGCCAATCCCCGTCCGAAGACATAAATGTCCAAGATGCCGGGGCAAAGACATCCGACTTCCCTCATGCGCCTCACTTGACTTTCTCGGCGCCTAAGTCTAGTACAGCTGAGACACGCACTCTACCAGGCCCGGTCCGACCACCTATTTCGGCAAACACCCTGGAAAACAACCTTCAGCCAGATGGTACCGGGATAGGGAAGCTACCACCCAATCATTGGCGCCTGCTGGATATCTACTTTTCCTACACGCATTCTTGGCTCCCTATCCTCGAGAAGAAAGACATGTACCAAGCATTGTACCAGTACTCTGAACAAGGCTCGTTGCTTCCCTCTGCGAATGTCGAGTCTGGCGTTCATGCCGAGCTCTGGAGCGCGCTCGCCCTGGCGTCCTTCCAGGCTGCTGCTACTGCTGCATCGAGTGCTACGGGTCCAGCTTCAGCTGCTCATGGCCATGACAATGCCATCAATCCTTCACCTGCAGACATATCTGACACAGCCCGAAAGCTCATACCTTTGGAAAGCGGGCCGTTCCAGGTTCAGCACTGCAGAGCGTTGCTGCTTCTTTGTCTCGTAAGCCTTGGGCGGGATGATTGGGAGTCTGCTTGGTTGCTGGTTGGCTTTGCGGTCCGCGTCCTACTTGTTGTTCGCACCCAGTTGCCTCCTGATGATGACCGGCCACGACCAAGAATGCGTGCGCTGCTCGTCGCGTGCTTCATCGTGGATACCATTGTGTCTATGAGACACAACGTGCCGGCCCATCTCAAGCCAGACGACATTGCGGATCTGCCGTTACCTGAAGACGGTCAAGATCAATGGGAGCCGTGGACACCATGTGAGGGCTTAGGCGGTGAACACACCATGCTGCAAATGTTGAGGAACCCGGCATACCCTTTAAGCACATTCAACCACCTATATGGCGTGACCAAGCTGGTTGCTTTGGAGCTTCTGCCAAGAATACGAACATCTTCACAGAACGCTCCCTTGGAGTTCAGGTCGCGGTTGCAGCAGGTAATCGGCCACAATTCTCCCTTCAGCGTCTTTGTCCTTTCCCAGGATACAGCATCGGCTTTTGTGCCTACTGCATACCTTACCCGTACCGTTTATTTATGGGCAGCTGCCTTTTCTGAGCCTCTCAACGAACACTACTCGCATCTTCTGATCGAAACTCTTGATCAGTATCAGAAGCGGTTTGGTACATATGCAATCCCACCTCTGATCCCTTCTCTTCTAGACTCCCTTCTTGCTTTAAAGAAACAATCACATTCTTCAGAGCGGCATCGAAGGCACTTGGAAGAGCTTTTCCCCGCCTACTCCTCCATTTGGCCTCGGGGAGGCCGACACAGCAATACTGGCCTCCAACCCATACGACAACTTGAGCTTCCGCCGACTGCGACTGCCACTGCAAGTATCATGCCCCATGTCATGGAACAGCCCCTGTCAACGTCAATAAATCCGGTCAATGATCGGTTTAATGGAATACCGAATCCTACCCCCTACAATAGCGATGCGGCCCTAGACGCAATTACTCAAACCAATGACTACGGATCAGTCAATACCCATGGCATTCTCAGTACATATCCACCACCTGCCACCCACTTGAACGAGGCCTCGGTGGCCCTTGCGCCTGGAGGTGCTCCTCCCAGACCACCCCCGCCATACGTTGATAGTACAACGAACCATCCTCCTTACCATAGTAACCTCGTCCCCATGGCCAATTTCGGATATTCGACTGTAGACTATGATGCGATGGTGGATGATCTGGCATCGATCGAGTACACGGATGCGGTTGACGTTGATCCGCAGTTCATGACGAACCTCGGGTTTGTCCCAGGGTGTAATTTTAGCGATATCAACACGTATGAGCAA

**Supplementary note 3: Gene sequences of *ZF9LOV^C1^-VP64***

In black: ZF9

In green: Linker

In orange: Tag

In blue: AsLOV2 (408-543)

In red: VP64

ATGGACTACAAGGACGACGATGACAAGCCCAAGAAAAAGCGCAAGGTCTCTAGACCCGGGGAGCGCCCCTTCCAGTGTCGCGCATCTTTGGAACGTATCGAAAAGAATTTTGTCATCACGGATCCGCGTCTTCCCGACAATCCGATTATCTTCGCGTCAGACTCTTTCTTACAACTGACTGAGTATAGTAGAGAGGAGATATTGGGGCGTAACTGTAGATTTCTTCAGGGGCCAGAAACTGATCGGGCTACCGTTCGCAAGATACGTGACGCAATAGACAACCAGACCGAGGTGACGGTGCAGCTGATTAACTACACAAAGTCTGGGAAGAAGTTCTGGAACCTGTTTCATTTACAACCTATGAGAGATCAAAAAGGTGACGTTCAATATTTCATCGGGGTTCAGTTAGATGGGACTGAGCACGTGAGAGATGCAGCAGAAAGAGAGGGTGTAATGCTTATTAAAAAAACAGCCGAGAATATCGACGAAGCCGCTGCGTCAATTTGCATGCGGAACTTTTCGGATAAAACTAAATTGAGAGTTCATACCCGTACTCATACCGGTGAAAAACCGTTTCAGTGTCGGATCTGTATGCGAAATTTCTCCGTTAGACATAATTTGACTAGACATCTACGTACGCACACCGGCGAGAAGCCATTCCAATGCCGAATATGCATGCGCAACTTCAGTCAATCTACTTCTTTGCAAAGACACCTAAAAACCCACCTGAGAGGATCCGGGCGCGCCGACGCGCTGGACGATTTCGATCTCGACATGCTGGGTTCTGATGCCCTCGATGACTTTGACCTGGATATGTTGGGAAGCGACGCATTGGATGACTTTGATCTGGACATGCTCGGCTCCGATGCTCTGGACGATTTCGATCTCGATATGTTAATTAATTGT

**Supplementary note 4: Codes for next-generation sequencing result analysis**

The original FASTQ files of all DNA sequencing results have been deposited in the Sequence Read Archive under the accession code PRJNA974403 [https://www.ncbi.nlm.nih.gov/sra/PRJNA974403].

The code is the same for all the sequencing results analysis, except for the FASTQ files to open. Here the codes for analysis of data with accession code SAMN35215818 [https://www.ncbi.nlm.nih.gov/biosample/35215818] was presented as an example.

Text files required for data analysis:

**Gal4VP64.log**

Atgaagctgctgagcagcatcgagcaggcctgtgacatctgccggctgaagaaactgaagtgcagcaaagaaaagcccaagtgcgccaagtgcctgaagaacaactgggagtgccggtacagccccaagaccaagagaagccccctgaccagagcccacctgaccgaggtggaaagccggctggaaagactggaacagctgtttctgctgatcttcccacgcgaggacctggacatgatcctgaagatggacagcctgcaggacatcaaggccctgctgaccggcctgttcgtgcaggacaacgtgaacaaggacgccgtgaccgacagactggccagcgtggaaaccgacatgcccctgaccctgcggcagcacagaatcagcgccaccagcagcagcgaggaaagcagcaacaagggccagcggcagctgacagtgtctgctgctgcaggcggaagcggaggctctggcggatctgatgccctggacgacttcgacctggatatgctgggcagcgacgccctggatgattttgatctggacatgctgggatctgacgctctggacgatttcgatctcgacatgttgggatcagatgcactggatgactttgacctggacatgctcggatcatag

**Gal4VP64_RC.log**

Ctatgatccgagcatgtccaggtcaaagtcatccagtgcatctgatcccaacatgtcgagatcgaaatcgtccagagcgtcagatcccagcatgtccagatcaaaatcatccagggcgtcgctgcccagcatatccaggtcgaagtcgtccagggcatcagatccgccagagcctccgcttccgcctgcagcagcagacactgtcagctgccgctggcccttgttgctgctttcctcgctgctgctggtggcgctgattctgtgctgccgcagggtcaggggcatgtcggtttccacgctggccagtctgtcggtcacggcgtccttgttcacgttgtcctgcacgaacaggccggtcagcagggccttgatgtcctgcaggctgtccatcttcaggatcatgtccaggtcctcgcgtgggaagatcagcagaaacagctgttccagtctttccagccggctttccacctcggtcaggtgggctctggtcagggggcttctcttggtcttggggctgtaccggcactcccagttgttcttcaggcacttggcgcacttgggcttttctttgctgcacttcagtttcttcagccggcagatgtcacaggcctgctcgatgctgctcagcagcttcat

**Gal4VP64_RC+9bp.log**

Ctatgatccgagcatgtccaggtcaaagtcatccagtgcatctgatcccaacatgtcgagatcgaaatcgtccagagcgtcagatcccagcatgtccagatcaaaatcatccagggcgtcgctgcccagcatatccaggtcgaagtcgtccagggcatcagatccgccagagcctccgcttccgcctgcagcagcagacactgtcagctgccgctggcccttgttgctgctttcctcgctgctgctggtggcgctgattctgtgctgccgcagggtcaggggcatgtcggtttccacgctggccagtctgtcggtcacggcgtccttgttcacgttgtcctgcacgaacaggccggtcagcagggccttgatgtcctgcaggctgtccatcttcaggatcatgtccaggtcctcgcgtgggaagatcagcagaaacagctgttccagtctttccagccggctttccacctcggtcaggtgggctctggtcagggggcttctcttggtcttggggctgtaccggcactcccagttgttcttcaggcacttggcgcacttgggcttttctttgctgcacttcagtttcttcagccggcagatgtcacaggcctgctcgatgctgctcagcagcttcatctatgatcc

**Gal4VP64+9bp.log**

atgaagctgctgagcagcatcgagcaggcctgtgacatctgccggctgaagaaactgaagtgcagcaaagaaaagcccaagtgcgccaagtgcctgaagaacaactgggagtgccggtacagccccaagaccaagagaagccccctgaccagagcccacctgaccgaggtggaaagccggctggaaagactggaacagctgtttctgctgatcttcccacgcgaggacctggacatgatcctgaagatggacagcctgcaggacatcaaggccctgctgaccggcctgttcgtgcaggacaacgtgaacaaggacgccgtgaccgacagactggccagcgtggaaaccgacatgcccctgaccctgcggcagcacagaatcagcgccaccagcagcagcgaggaaagcagcaacaagggccagcggcagctgacagtgtctgctgctgcaggcggaagcggaggctctggcggatctgatgccctggacgacttcgacctggatatgctgggcagcgacgccctggatgattttgatctggacatgctgggatctgacgctctggacgatttcgatctcgacatgttgggatcagatgcactggatgactttgacctggacatgctcggatcatagatgaagctg

Here is the MATLAB script

function strout = reverse_complement(strin)

strin = upper(strin);

strout = strin;

inds = find(strin == 'A');

strout(inds) = 'T';

inds = find(strin == 'C');

strout(inds) = 'G';

inds = find(strin == 'G');

strout(inds) = 'C';

inds = find(strin == 'T');

strout(inds) = 'A';

strout = strout(end:-1:1);

end

% INPUT FILE:

fid_in = fopen('S1_read1.fastq');

fid_in2 = fopen('S1_read2.fastq');

% OUTPUT FILES: These files are created when you run this code

fid_left = fopen('reads_left.log', 'w');

fid_right = fopen('reads_right.log', 'w');

% SEQUENCES TO LOOK FOR:

left_seq = lower('TGCATCTT');

left_RC = lower(reverse_complement(left_seq));

right_seq = lower('TGACGCAG');

right_RC = lower(reverse_complement(right_seq));

% Number of bases to check against plasmid sequence

Nmatch = 10;

%% Run the loop!

c = 0;

while 1

c = c + 1;

line = lower(fgetl(fid_in));

if isnumeric(line) && line == -1

break

end

% if c > 1e5, break

if mod(c,4) == 2

match = regexp(line, left_RC, 'once');

if ~isempty(match)

line = lower(reverse_complement(line));

end

match = regexp(line, left_seq, 'once');

if match > Nmatch

fprintf(fid_left, '%s\n', line((match-Nmatch):(match-1)));

end

match = regexp(line, right_RC, 'once');

if ~isempty(match)

line = lower(reverse_complement(line));

end

match = regexp(line, right_seq, 'once');

if match > Nmatch

fprintf(fid_right, '%s\n', line((match-Nmatch):(match-1)));

end

end

end

c = 0;

while 1

c = c + 1;

line = lower(fgetl(fid_in2));

if isnumeric(line) && line == -1

break

end

% if c > 1e5, break

if mod(c,4) == 2

match = regexp(line, left_RC, 'once');

if ~isempty(match)

line = lower(reverse_complement(line));

end

match = regexp(line, left_seq, 'once');

if match > Nmatch

fprintf(fid_left, '%s\n', line((match-Nmatch):(match-1)));

end

match = regexp(line, right_RC, 'once');

if ~isempty(match)

line = lower(reverse_complement(line));

end

match = regexp(line, right_seq, 'once');

if match > Nmatch

fprintf(fid_right, '%s\n', line((match-Nmatch):(match-1)));

end

end

end

%% Plasmid sequence

fid = fopen('Gal4VP64+9bp.log');

plasmid_and_9bp = lower(fgetl(fid));

fclose(fid);

fid = fopen('Gal4VP64.log');

plasmid = lower(fgetl(fid));

fclose(fid);

fid = fopen('Gal4VP64_RC.log');

plasmid_RC = lower(fgetl(fid));

fclose(fid);

fid = fopen('Gal4VP64_RC+9bp.log');

plasmid_9bp_RC = lower(fgetl(fid));

fclose(fid);

% These are the vectors of where a match appears!

plasmid_sites_left = zeros(size(plasmid)); % initialize to zeros

plasmid_sites_right = zeros(size(plasmid)); % initialize to zeros

%% First, find matches for all the LEFT transposons

fid = fopen('reads_left.log');

c = 0;

while 1

c = c + 1;

% Read 1 line from the file

line = fgetl(fid);

if isnumeric(line) && line == -1

break

end

[first,match] = regexp(plasmid_and_9bp, lower(line),'once');

if ~isempty(match) && match <= length(plasmid)

plasmid_sites_left(match) = plasmid_sites_left(match)+1; % add 1 hit in the vector of plasmid integration sites at the 'match' position

end

if ~isempty(match) && match > length(plasmid)

plasmid_sites_left(match-length(plasmid)) = plasmid_sites_left(match-length(plasmid))+1;

end

end

fclose(fid);

fclose all;

sum_plasmid_sites_left = sum(plasmid_sites_left);

%% Then, find matches for all the RIGHT transposons

fid = fopen('reads_right.log');

c = 0;

while 1

c = c + 1;

% Read 1 line from the file

line = fgetl(fid);

if isnumeric(line) && line == -1

break

end

[first,last] = regexp(plasmid_9bp_RC, lower(line),'once');

if ~isempty(last) && length(plasmid)-(last-4)+1 > 0

transformindex = length(plasmid)-(last-4)+1;

plasmid_sites_right(transformindex) = plasmid_sites_right(transformindex)+1; % add 1 hit in the vector of plasmid integration sites at the 'match' position

end

if ~isempty(last) && length(plasmid)-(last-4)+1 <= 0

transformindex = 2*length(plasmid)-(last-4)+1;

plasmid_sites_right(transformindex) = plasmid_sites_right(transformindex)+1;

end

end

fclose(fid);

fclose all;

sum_plasmid_sites_right = sum(plasmid_sites_right);

plasmid_sites_right_transpose = transpose(plasmid_sites_right);

plasmid_sites_right_inframe_transpose = plasmid_sites_right_transpose(2:3:end,:);

plasmid_sites_right_inframe = transpose(plasmid_sites_right_inframe_transpose);

plasmid_sites_left_transpose = transpose(plasmid_sites_left);

plasmid_sites_left_inframe_transpose = plasmid_sites_left_transpose(2:3:end,:);

plasmid_sites_left_inframe = transpose(plasmid_sites_left_inframe_transpose);
