# Supplementary material for: Light-switchable transcription factors obtained by direct screening in mammalian cells
Source: Nat Commun. 2023 Jun 2;14:3185. doi: 10.1038/s41467-023-38993-6 (PMC10238501; doi:10.1038/s41467-023-38993-6)
Supplement: Supplementary file 2 — Reporting Summary [file 41467_2023_38993_MOESM2_ESM.pdf]

nature portfolio

Corresponding author(s): Jared Toettcher

Last updated by author(s): May 22, 2023



# Reporting Summary

Nature Portfolio wishes to improve the reproducibility of the work that we publish. This form provides structure for consistency and transparency in reporting. For further information on Nature Portfolio policies, see our Editorial Policies and the Editorial Policy Checklist.

## Statistics

For all statistical analyses, confirm that the following items are present in the figure legend, table legend, main text, or Methods section.

| n/a | Confirmed | |
|---|---|---|
| ☐ | ☒ | The exact sample size ($n$) for each experimental group/condition, given as a discrete number and unit of measurement |
| ☐ | ☒ | A statement on whether measurements were taken from distinct samples or whether the same sample was measured repeatedly |
| ☒ | ☐ | The statistical test(s) used AND whether they are one- or two-sided<br>*Only common tests should be described solely by name; describe more complex techniques in the Methods section.* |
| ☒ | ☐ | A description of all covariates tested |
| ☒ | ☐ | A description of any assumptions or corrections, such as tests of normality and adjustment for multiple comparisons |
| ☐ | ☒ | A full description of the statistical parameters including central tendency (e.g. means) or other basic estimates (e.g. regression coefficient) AND variation (e.g. standard deviation) or associated estimates of uncertainty (e.g. confidence intervals) |
| ☒ | ☐ | For null hypothesis testing, the test statistic (e.g. $F$, $t$, $r$) with confidence intervals, effect sizes, degrees of freedom and $P$ value noted<br>*Give P values as exact values whenever suitable.* |
| ☒ | ☐ | For Bayesian analysis, information on the choice of priors and Markov chain Monte Carlo settings |
| ☒ | ☐ | For hierarchical and complex designs, identification of the appropriate level for tests and full reporting of outcomes |
| ☒ | ☐ | Estimates of effect sizes (e.g. Cohen's $d$, Pearson's $r$), indicating how they were calculated |

*Our web collection on statistics for biologists contains articles on many of the points above.*

## Software and code

Policy information about availability of computer code

| | |
|---|---|
| Data collection | SH800S Cell Sorter Software was used for collecting flow cytometry data. |
| Data analysis | We used MATLAB R2021b and custom MATLAB scripts for analyzing next-generation sequencing data. Codes are provided in the Supplementary information. |

For manuscripts utilizing custom algorithms or software that are central to the research but not yet described in published literature, software must be made available to editors and reviewers. We strongly encourage code deposition in a community repository (e.g. GitHub). See the Nature Portfolio guidelines for submitting code & software for further information.

## Data

Policy information about availability of data

All manuscripts must include a data availability statement. This statement should provide the following information, where applicable:

- Accession codes, unique identifiers, or web links for publicly available datasets
- A description of any restrictions on data availability
- For clinical datasets or third party data, please ensure that the statement adheres to our policy

There are no restrictions on availability. All raw data generated in this study are provided in the Supplementary Information/Source Data file. The original FASTQ files of all DNA sequencing results have been submitted to Sequence Read Archive under the accession code PRJNA974403 [https://www.ncbi.nlm.nih.gov/sra/PRJNA974403]. All plasmids and cell lines presented in this study will be shared upon reasonable request from the corresponding author.

## Human research participants

Policy information about studies involving human research participants and Sex and Gender in Research.

| | |
|---|---|
| Reporting on sex and gender | N/A |
| Population characteristics | N/A |
| Recruitment | N/A |
| Ethics oversight | N/A |

Note that full information on the approval of the study protocol must also be provided in the manuscript.

# Field-specific reporting

Please select the one below that is the best fit for your research. If you are not sure, read the appropriate sections before making your selection.

- [x] Life sciences
- [ ] Behavioural & social sciences
- [ ] Ecological, evolutionary & environmental sciences

For a reference copy of the document with all sections, see nature.com/documents/nr-reporting-summary-flat.pdf

# Life sciences study design

All studies must disclose on these points even when the disclosure is negative.

| | |
|---|---|
| Sample size | During the high-throughput sorting, we sorted a large excess of cells to ensure at least 100-fold representation of all insertion sites. For transcription assay we measured many thousands of cells in each flow cytometry analysis in multiple biological replicates. No sample sizes calculations were performed. We chose to do three biological replicates for the characterization of LightsOut, which is sufficient to demonstrate the light-switchable behavior of LightsOut due to small standard deviation between groups in the same condition and significant difference in result between groups in dark and in light. For the characterization of other light-switchable proteins like LOV-inserted QF and LOV-inserted ZF9, either two or three biological replicates were performed for each condition. When three biological replicates were performed, clear conclusion could be drawn due to small standard deviation between groups in the same condition and clear comparison in result between groups in dark and in light. When two biological replicates were performed, clear conclusion in turns of light-switchable behavior could still be drawn due to significant difference in result between groups in dark and in light. All the experiments include a wild type negative control whose transcription performance has no difference between light and dark. Moreover, we characterized the performance not only in the context of simple transfection but also in the context of stable cell line and sorted library, all of which gave rise to the same result and conclusion. Overall, we think the sample sizes we chose for transcription assay are enough to support our conclusion. |
| Data exclusions | None. |
| Replication | The high-throughput cell sorting was performed only once since it's very time-consuming. All other experiments were repeated at least twice with similar results, and the number of replicates for each condition or experiment was stated in main text and supplementary information. All attempts at replication were successful. |
| Randomization | Different conditions were randomly assigned to the wells on a 12-well plate. |
| Blinding | No blinding was performed. Since every experiment would end up with a specific number given by instruments unbiasedly as the result for following analysis, we don't think performing blinding is necessary. |

# Reporting for specific materials, systems and methods

We require information from authors about some types of materials, experimental systems and methods used in many studies. Here, indicate whether each material, system or method listed is relevant to your study. If you are not sure if a list item applies to your research, read the appropriate section before selecting a response.

## Materials & experimental systems

| n/a | Involved in the study | |
|---|---|---|
| ☒ | ☐ | Antibodies |
| ☐ | ☒ | Eukaryotic cell lines |
| ☒ | ☐ | Palaeontology and archaeology |
| ☒ | ☐ | Animals and other organisms |
| ☒ | ☐ | Clinical data |
| ☒ | ☐ | Dual use research of concern |

## Methods

| n/a | Involved in the study | |
|---|---|---|
| ☒ | ☐ | ChIP-seq |
| ☐ | ☒ | Flow cytometry |
| ☒ | ☐ | MRI-based neuroimaging |

# Eukaryotic cell lines

Policy information about cell lines and Sex and Gender in Research

| | |
|---|---|
| Cell line source(s) | HEK293T, SUM159, and NIH3T3 cells. 293T-LP cell line is derived from HEK293T cells where one copy of landing pad sequence was integrated into the genome, and was received as a gift from Dr. Kenneth Matreyek (Case Western Reserve University), which had been bought from ThermoFisherScientific. SUM159s were obtained as a gift from Dr. Yibin Kang (Princeton Univ.). NIH3T3 cells were received as a gift from Dr. Wendell Lim (UCSF). |
| Authentication | NIH3T3 cells were authenticated by STR profiling. It is important to note that we do not draw biological conclusions about any cell lines - they are merely a chassis for testing our light-activated gene expression circuit, which functions equivalently in all cell lines tested. |
| Mycoplasma contamination | We regularly test for mycoplasma and no cell lines were contaminated. |
| Commonly misidentified lines (See ICLAC register) | No commonly misidentified cell lines were used in the study |

# Flow Cytometry

## Plots

Confirm that:

☒ The axis labels state the marker and fluorochrome used (e.g. CD4-FITC).

☒ The axis scales are clearly visible. Include numbers along axes only for bottom left plot of group (a 'group' is an analysis of identical markers).

☒ All plots are contour plots with outliers or pseudocolor plots.

☒ A numerical value for number of cells or percentage (with statistics) is provided.

## Methodology

| | |
|---|---|
| Sample preparation | Final GFP levels were characterized by flow cytometry. For analysis, single cells were gated using FSC-A and SSC-A (for live cells) and FSC-A and FSC-H (for single cells). See Figure S9 for gating strategy. |
| Instrument | Sony SH800 cell sorter |
| Software | Sony flow cytometry software |
| Cell population abundance | Forward scatter / side scatter gating was used to identify live cells, with a typical abundance of 60-70% passing the gate (see Figure S9). No other gating was performed for analysis. Light/dark selection was performed using gates for high and low GFP (see Figure S2). |
| Gating strategy | Figure S9 shows our gating strategy along with abundance in each population. |

☒ Tick this box to confirm that a figure exemplifying the gating strategy is provided in the Supplementary Information.
