# Supplementary figures and images for: Light-switchable transcription factors obtained by direct screening in mammalian cells
Source: Nat Commun. 2023 Jun 2;14:3185. doi: 10.1038/s41467-023-38993-6 (PMC10238501; doi:10.1038/s41467-023-38993-6)

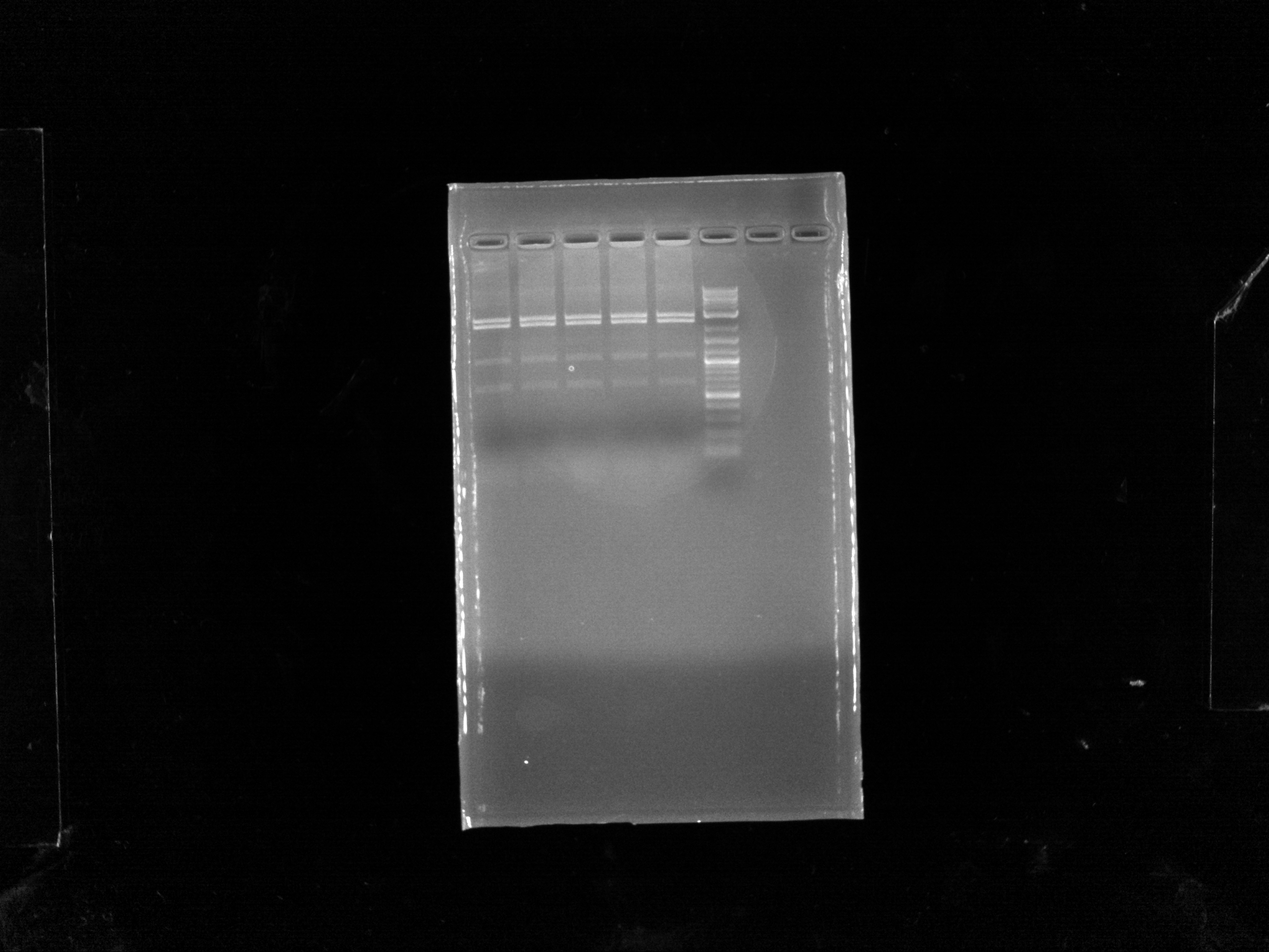

Supplement: Supplementary file 4 — Source Data [file 41467_2023_38993_MOESM4_ESM.zip › Source data fig. 1d.tif]
